# The views of health guideline developers on the use of automation in health evidence synthesis

**DOI:** 10.1186/s13643-020-01569-2

**Published:** 2021-01-08

**Authors:** Anneliese Arno, Julian Elliott, Byron Wallace, Tari Turner, James Thomas

**Affiliations:** 1 EPPI-Centre, UCL Social Science Research Institute, University College London, London, UK; 2 School of Public Health and Preventive Medicine, Monash University, Melbourne, Australia; 3 Khoury College of Computer Sciences, Northeastern University, Boston, USA

**Keywords:** Automation, Systematic reviews, Machine learning, Guideline development, Diffusion of Innovation, Evidence synthesis

## Abstract

**Background:**

The increasingly rapid rate of evidence publication has made it difficult for evidence synthesis—systematic reviews and health guidelines—to be continually kept up to date. One proposed solution for this is the use of automation in health evidence synthesis. Guideline developers are key gatekeepers in the acceptance and use of evidence, and therefore, their opinions on the potential use of automation are crucial.

**Methods:**

The objective of this study was to analyze the attitudes of guideline developers towards the use of automation in health evidence synthesis. The Diffusion of Innovations framework was chosen as an initial analytical framework because it encapsulates some of the core issues which are thought to affect the adoption of new innovations in practice. This well-established theory posits five dimensions which affect the adoption of novel technologies: *Relative Advantage*, *Compatibility*, *Complexity*, *Trialability*, and *Observability*. Eighteen interviews were conducted with individuals who were currently working, or had previously worked, in guideline development. After transcription, a multiphase mixed deductive and grounded approach was used to analyze the data. First, transcripts were coded with a deductive approach using Rogers’ Diffusion of Innovation as the top-level themes. Second, sub-themes within the framework were identified using a grounded approach.

**Results:**

Participants were consistently most concerned with the extent to which an innovation is in line with current values and practices (i.e., *Compatibility* in the Diffusion of Innovations framework). Participants were also concerned with *Relative Advantage* and *Observability*, which were discussed in approximately equal amounts. For the latter, participants expressed a desire for transparency in the methodology of automation software. Participants were noticeably less interested in *Complexity* and *Trialability*, which were discussed infrequently. These results were reasonably consistent across all participants.

**Conclusions:**

If machine learning and other automation technologies are to be used more widely and to their full potential in systematic reviews and guideline development, it is crucial to ensure new technologies are in line with current values and practice. It will also be important to maximize the transparency of the methods of these technologies to address the concerns of guideline developers.

**Supplementary Information:**

The online version contains supplementary material available at 10.1186/s13643-020-01569-2.

## Background

### Evidence-based guidelines are overwhelmed by the rate of research publication

As guidelines increasingly incorporate an evidence-based medicine approach, the systematic reviews which are a crucial component of this evidence have become overwhelmed by the rate of publication of new evidence [1]. With nearly 4000 health research articles published daily, systematic reviews cannot keep up with the deluge of data [2]. Research is at risk of being wasted, leading to out-of-date healthcare and guidelines and consequently impacting on population health outcomes.

### Limited literature addresses adoption of automation

Given this, there is increasing interest in the use of automation in the completion of systematic reviews [3–6]. While some herald the use of automation, others are hesitant to adopt these novel methods. Automation technologies include machine learning (ML), natural language processing (NLP), and text mining, among other technologies.

The literature on the topic of automation in health evidence synthesis is somewhat lacking. Previous publications largely focus on two main areas: potential applications of automation and the validity or accuracy of automation tools [5]. The uptake of automation has been notably slow [6, 7], despite the broad availability of various tools, particularly for study screening, and the availability of peer-reviewed literature addressing the accuracy and potential integration opportunities. This leads to the question: what factors are inhibiting the uptake of automation into systematic reviews and into guidelines?

There has not yet been significant primary research into barriers and facilitators to uptake of automation in health evidence synthesis contexts [7, 8]. Considering the slow adoption rate, understanding the barriers and facilitators to uptake is important, as is exploring the perceptions of key stakeholders in evidence production towards the uptake of automation. A range of groups could be considered stakeholders on this topic, including patients, caregivers, healthcare professionals, researchers, or guideline developers. As outlined above, guidelines are a key component in the translation of knowledge to practice; therefore, evidence from guideline developers detailing their views of the use of automation would be helpful in elucidating the reasons for the slow adoption of automation.

### Diffusion of Innovations

Rogers’ Diffusion of Innovations is a highly applicable framework for the analysis of these views and the adoption of automation in health evidence production more broadly [9]. This theory describes how, why, and at what rate an innovation spreads, the characteristics of the innovation that play a role in this process, and the typical categories of innovators. Its insights have been repeatedly supported by empirical data in a broad range of contexts.

The characteristics of an innovation that impact its diffusion are described as follows:

1. Complexity: *Complexity* is how easy an innovation is, or is perceived to be, to comprehend and to put into use.
2. Compatibility: *Compatibility* refers to an innovation being in line with existing values and practices, and the needs of potential future users.
3. Trialability: *Trialability* refers to the ability of users or potential users to experiment with an innovation prior to adopting it.
4. Observability: *Observability* is the degree to which potential users may examine the results of an innovation.
5. Relative Advantage: *Relative Advantage* refers to how much better an innovation is, or is perceived to be, than the system it is replacing.

These five elements collectively influence potential adopters’ decisions towards the adoption of an innovation. In distinct contexts, and with distinct populations, some characteristics may play a greater role than some others. For example, farmers considering whether to adopt a new system of irrigation may hold different values from pilots considering a new autopilot software. Understanding the comparative role of these characteristics in the context of systematic reviews’ and health guidelines’ potential use of automation should prove useful in describing the current state of adoption, as well as considering future research foci and organizational norm-setting.

### Research questions

The goal of this research was to gather data from guideline developers regarding their attitudes and perceptions of automation, and specifically automation applied to health evidence production. Research questions were as follows:

1. To what extent do the opinions of guideline developers of automation of health evidence synthesis fit into the Diffusion of Innovations framework?
2. Within the Diffusion of Innovations themes, what important concepts were identified by participants?

## Methods

Ethical approval was granted from the University College London Institute of Education prior to commencement of the study.

### Participants

Participants were invited to participate by email sent to existing personal and professional networks for guideline developers and systematic review researchers. These networks included but were not limited to Guidelines International Network (GIN), National Institute for Health Care and Excellence (NICE, UK), and the National Health and Medical Research Council (NHMRC, Australia). Participants were required to be developers of health policy or clinical practice guidelines and/or to have firsthand experience with guideline development. Participants whose experience was limited to systematic reviews or other research not including guideline development were excluded.

Potential participants were invited to participate in a semi-structured one-on-one interview conducted via phone or via Skype with Ms. Arno, with the session to be audio recorded with participant consent. None personally knew Ms. Arno prior to the completion of the interviews. Participants were provided information on her background as a PhD student at the University College London studying the adoption of automation in health evidence synthesis. An interview instrument was developed by the lead author and validated by the research team prior to the interviews and was applied in all interview sessions, with variation in follow-up questions as relevant according to participants’ responses.

### Data collection and analysis

Following verbatim transcription of the interviews, they were provided to the participants for validation. They were then analyzed using QSR NVivo 12 [10].

A thematic approach, as outlined by Braun et al. [11], was applied. This method was adapted to incorporate both deductive and inductive analyses. This allowed for framework analysis in addition to reflexive and iterative insights from the resulting data [12].

The analysis took place in five stages, described in more detail below.

#### Stage 1: Assignment within predefined frameworks

First, Rogers’ Diffusion of Innovation framework was used as the top-level deductive codes, using a line-by-line verbatim assignment of transcripts to one or more of the five themes (i.e., *Complexity*, *Compatibility*, *Trialability*, *Observability*, and *Relative Advantage*).

#### Stage 2: Open coding within the Diffusion of Innovations framework

Once each transcript was coded according to the top-level frameworks (i.e., Diffusion of Innovations), a codebook—a document containing all data belonging to a code or theme—was generated for each of the five themes. These codebooks were then examined with an open coding method.

#### Stage 3: Generation of themes

The codebook of each Diffusion of Innovation theme was reviewed across all transcripts together to identify the shared patterns among the grounded open codes. Each individual verbatim code was grouped with others with similar meaning and content, forming preliminary explanatory themes.

Following the formation of these themes, a further review process was undertaken to reconsider how the themes fitted together. In addition, outlying codes were identified as those that either had not been grouped with codes from other transcripts or those that had relatively few grounded codes grouped together.

#### Stage 4: Generation of matrices

A matrix was generated comparing each of the top-level framework themes against the data-driven themes. This approach not only allowed for an overview of the relative significance of each overall theme—thus addressing research question 1—but also to examine this significance through different lenses. For instance, isolating the framework to selected grounded codes might give a different impression of the results of the framework analysis.

#### Stage 5: Identifying patterns and outliers

These matrices were finally used to analyze the data in relation to the first research question (i.e., how do guideline developers’ opinions on automation relate to the Diffusion of Innovations framework?) and to expand upon these data in relation to the second research question (i.e., what important concepts were identified by participants?).

## Results

### Participants

Twenty individuals responded to the email invitations. Eighteen interviews were conducted and varied in length from approximately 30 min to approximately 80. The remaining two respondents were deemed ineligible due to the lack of firsthand guideline development experience. Five participants were male, and 13 were female. Half of the participants had between 5 and 10 years of experience in evidence synthesis; five participants had between 10 and 20 years of experience; two participants had more than 20 years of experience, and two had less than 5 years of experience. Ten participants’ current primary affiliation was with a university, seven with a government body, and one in the private sector. Eleven participants were based in Australia, with the remaining seven based either in the USA or in the UK. In addition to NICE and NHMRC, organizations represented included the Agency for Healthcare Research and Quality (AHRQ, USA), the Joanna Briggs Institute (JBI, Australia), the World Health Organization (WHO), and private consultancies. No participants withdrew from the study, and no repeat interviews were required.

### Overview

Interview transcripts demonstrated high consistency in the distribution within the Diffusion of Innovations thematic framework. Following initial coding (stage 1), *Compatibility* was the most prominently discussed theme across all participants. *Relative Advantage* and *Observability* were also given substantial attention from participants’ discussions, though to a lesser extent than *Compatibility*. *Trialability* and *Complexity* were the least discussed themes among all participants.

### Compatibility

All participants discussed their values as guideline developers at length, both within the context of the potential use of automation and independent from it. Emphasis on the *Compatibility* theme was consistently far stronger than any of the other four themes in the deductive framework being applied in this analysis. Some examples of values were a “rigorous” approach to the evaluation of evidence and careful construction of questions. Relating to automation more specifically, participants highlighted a need for human and organizational involvement.

How you synthesize it, how you pull it together is kind of key. Participant 3

I think it would be a shame if humans weren’t involved in [synthesis]. Participant 9

Two sub-themes were identified within the *Compatibility* theme which further detailed participants’ desire to match new practices with the values which underpin current practices: *ability to double-check* and *transparency as accountability*.

### Ability to double-check

Most participants indicated the importance of the *ability to double-check* the output of automation with human researcher input. These discussions often cited the rationale that current practices usually involve a human double-checking the work of another human and posited that newer workflows should therefore maintain this pattern.

Some, but not all, participants indicated that reproducibility was the underlying reason for the double-checking status quo. It is possible that views of participants would be different should rigorous research alter overall perceptions of the reproducibility of automated screening and extraction; this is further discussed as a contextual factor in subsequent sections.

I can see it could be done. But surely it would need to be checked by someone anyway. Because even if it’s done by a human with vast experience, it’s always important to have a second person to check it. Participant 5

At the minute the standard is for two operators. So you’d want it to have been checked by a second method, if not person. So that would be my only thing – reproducibility. Participant 7

### Transparency as accountability

Several participants wanted to ensure that any automation methods used in synthesizing evidence were freely accessible and transparent to examination. Many emphasized that they are accountable to stakeholders who need to be sure they have not missed any information, and therefore require the ability to freely examine methods used, including any automation.

The trustworthiness of evidence in general is integral to the professional culture of guideline development and was emphasized by the participants. Trustworthiness and methods to verify it therefore extend to new tools that use automation, in the view of participants, in the form of transparency and validation.

A group of experts can apply judgement to that body of evidence, and needs to know they can trust the evidence that you’d found. Participant 12

The key part of working with a face to face committee … Is you have they have to have total confidence in what the technical team has done. Participant 16

### Relative Advantage

When discussing *Relative Advantage*, participants focused on the *freeing up of human resources*, and to a lesser extent on *time and cost saving*. When prompted to discuss ML directly (in contrast to general views of evidence synthesis and guideline development approaches), participants tended to more frequently discuss ideas relating to the *Relative Advantage* of automation. Participants were interested in freeing time and money, but contingent upon the automation perfectly matching perceived human quality.

### Freeing up human resources

The primary advantage specified in the discussion with participants was the potential to free up human resources for rededication to additional tasks within the health evidence ecosystem.

In research time is always limited and you know there’s never enough grant money to help employ staff … by having a machine do it, it would be cost-effective, and spare the researchers’ time to do other research-related tasks. Participant 17

### Time and cost saving

Some participants also identified that automation might potentially save time and/or save money. Strikingly, no participants indicated an openness to any trade-off between accuracy and time.

No matter how quickly a guideline’s done, everybody always wants it faster and to be of high quality. So anything that can improve on that would be welcome, I think. Participant 11

### Observability

Participants communicated that they would like to see evidence prior to implementing new practices, as well as a sustained ability to cross-examine the behavior of the technologies.

### Need for evidence

The need for rigorously produced, disseminated, and easily accessed evidence was clear in the data. Several participants expressed an openness to automation being integrated into evidence synthesis, on the condition that accuracy has been demonstrated.

I think at the moment it has a potentially high level of risk of being incorrect. But I don’t really know enough about it. I’d need to be convinced about it I think to consider it. Participant 9

If the whole process were done by some machine or machine learning application, I think it would need to be properly trialed. Participant 5

As long as there was clear data to support that … machine-learning is a reliable method, but you know, better than or equal to humans doing it. Participant 17

One notable outlier indicated they were already convinced of automation’s abilities within the specific context of screening. This unusual case raises the possibility that these results would be different given further evidence production and dissemination.

I do think it’s been well demonstrated for the screening aspects, for the hit rates of what gets included and what doesn’t, and how correct it is. Participant 11

### Personal need for double-checking

Participants often wanted an established and ongoing method of observing the inner workings of the ML processes, frequently described as a desire to “check” what ML had done. While similar to the previous theme of *Compatibility: ability to double-check*, the latter discussed that guideline developers believe the ability to check methods should be available as a matter of principle, while *Observability: personal need for double-checking* discusses that guideline developers want to do such checking themselves.

This need to be able to continually check how the machine learning has processed information could be interpreted as a desire to maintain control over the evidence synthesis process. As previously discussed, guideline developers must convince other stakeholders of their recommendations’ integrity, so personal quality control fits in with the cultural expectations of guideline development.

The thing that’s sort of a little bit distressing from a novice point of view with machine-learning is not feeling like I have a way to check it… I’d need some way to be confident …. [I’d need] a way to check the algorithms. Participant 3

### Complexity and Trialability

Selected participants identified that the learning process would need to be simple if researchers were to adopt ML. They also expressed a preference for familiarity over the novel.

Whenever you try and really change things, I think there’s a degree of skepticism anyway…I think that might just be the nature of human beings. Participant 9

If they have to learn the process, and if it’s hard, then that sort of discourages them. Participant 18

So unless the technology offers a value add that’s substantial enough to overcome the learning curve…however much time it takes to do that has to not be more time than you’re gonna save. Participant 3

### Contextual themes

Upon re-examination of how the data informed the deductive framework (described in step 5 of the “Methods” section), several contextual factors were identified.

### Participant familiarity with automation

Participants nearly always offered disclaimers prior to commenting, indicating they felt they did not have sufficient experience with automation technologies to be able to comment at their desired level of expertise. These data were of significant interest as they demonstrated a current lack of robust knowledge of the capabilities of automation within the target population.

I’ve done a very little bit with machine-learning. Participant 3

It’s just my concern would be that I’ve not had any experience with it. Participant 7

I haven’t had much to do with machine-learning. Like I’ve kind of heard about it. Participant 17

I think that’s something I have no personal experience with. Participant 11

To be honest I actually haven’t had much experience with it. Participant 8

Yeah, I don’t know, I don’t really understand that process. Participant 5

### Overall skepticism towards machine learning

Overall skepticism or mistrust towards automation, both towards current technologies and anticipated future ones, was clear in the contributions from participants. They particularly expressed doubt over the ability of a machine to mimic human judgment calls they felt are currently essential to well-formulated health guidelines.

It would be very difficult to train a machine to make the sort of value decisions that we have to make. Participant 10

I’m still a bit nervous about some of the interpretation of that…it just might be a distrust about it, I think? Participant 13

How can a computer apply judgement? …There’s judgement required when it comes to things like quality or – they are not things I expect to be evidence that could be accurate. Participant 12

I don’t think it could fully replace a human … I think there can be subtleties between how things can interact… I think there’s always going to be some sort of human element. Participant 9

I don’t know if we’re there yet. Maybe we’ll get to the point where we can do that, but to do that, like quality rating, or to do a level of evidence, or strength of evidence… I mean there’s still a lot of value judgements in that. And I don’t know how much machine learning could help with that at this point. Participant 3

**Fig. 1:**
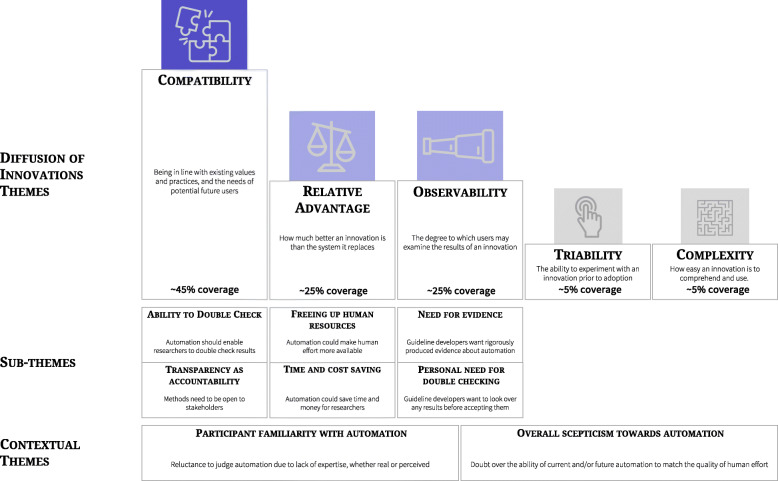
Summary of results

Figure 1 presents a summary of the themes, sub-themes, and contextual themes.

## Discussion

### Cultural standards of practice greatly influence decision-making

Guideline developers demonstrated deeply held core beliefs about evidence synthesis methodology and perceived quality. These will be central in the potential adoption of automation to health evidence synthesis.

A 2013 paper commenting on the reasons for the slow uptake of automation posed the broad question: “why is [automation] not yet widely used?” [13]. At the time, the authors concluded that “further technical and empirical work is needed … [to] develop solutions which have a demonstrative relative advantage, and which are clearly compatible with the needs of systematic reviewers and their users.” That is, they considered *Relative Advantage* and *Compatibility* the most important themes, playing the most significant roles in the adoption of automation. Considering the data presented in this study in relation to Thomas’ [13] question, the prior conclusions should be adjusted slightly.

The most significant reason appears to be that key stakeholders of EBM are not persuaded that automation is compatible with their guiding values, principles, or standards. While *Relative Advantage* was important, it was secondary to the far more prominent discussion of *Compatibility*. Further, the identified sub-themes of *Compatibility* focused more on automation’s fitting in with the values behind current practices, rather than on fitting in with existing infrastructure

The preceding points not only represent a shift from the hypotheses presented in the literature, but also from the focus of previous discussions at relevant conferences. The International Collaboration for the Automation of Systematic Reviews (ICASR), formed in 2015, is a global network endeavoring to successfully integrate all the parts of automation of systematic review production together. In the notes of the third ICASR meeting in 2017, the group concluded that the “most pressing needs at present are to develop approaches for validating” automation and integration with existing system architecture [14]. Stated another way, ICASR believed *Observability* to be critical to uptake, as well as *Compatibility* specifically in reference to fitting into existing practice.

As in the previous case discussed, this research has provided some evidence to support this assertion but suggests a slight redirection in which priorities would be best suited to the promotion of automation adoption. While evidence gathered in this study reinforces that *Compatibility* plays a significant role, it also demonstrates that alignment with values is more highly prioritized than alignment with current practice and system architecture. In addition, the “most pressing need” may not be validation (*Observability*), but instead the demonstration and communication of methodological standards and cultural coordination (*Compatibility*).

### Researcher effort will be redirected rather than replaced

Guideline developers anticipate that automation will be most useful in redirecting person-time rather than replacing it. They highlighted that a critical (and in their view, irreplaceably human) part of their professional contribution is the nuanced judgments applied to the presented evidence, often derived from lived experience. Automation could contribute to an improvement in guideline quality by providing additional resources (namely, time) to more difficult aspects of guideline development, and not simply by cutting costs and workload.

Contributions from participants in this study relating to the reluctance to relinquish human judgment align with notes from the previously mentioned ICASR meeting [14]. They stated:

For example, external stakeholders might believe the current vision is automated reviews devoid of valuable human control and input, that is, a general autonomous artificial intelligence system. That view, however, was neither represented nor sanctioned at the meeting. Therefore, improving the terminology associated with systematic review automation to reflect the goal more accurately is likely valuable.

This study provides evidence in support of this proposition: participants were wary of automation in part due to the idea that it might remove crucial human judgment in the process of guideline development; notably, encouraging complete and total replacement is “neither represented nor sanctioned.” Given that participants in this study echoed this fear, it raises the question of why guideline developers hold this view, and how to best communicate a more accurate representation of the goals of promoters of automation in systematic reviews and guidelines.

This observed anticipation of automation allowing for refocusing of effort is what should be expected if the results of this study are situated in the historical evidence and context. From the late nineteenth century onwards, there have been repeated waves of automation of production and consequent population-level job panic [15]. With each wave, however, human effort has not been removed, but rather redirected. In some cases, job opportunities have expanded rather than contracted as a by-product of widespread automation. Therefore, in addition to enabling the valuable skills of EBM researchers to be better spent, it is possible the field will see an expansion of opportunities.

As in the previous section, perhaps proponents of automation have a choice to steer the general conversation to clarify that expert opinion will not be superseded but will instead be made less costly and more available by freeing up person-time and other resources. An enabling environment for the promotion and adoption of automation in a manner that redirects rather than replaces research effort could be an effective strategy in building consensus among guideline developers, as key stakeholders of the evidence synthesis process, in accepting, implementing, and promoting automation practices.

## Study limitations

### Participant sample

A potential limitation of a study of this kind is the construction of the sample and whether a different sample might lead to different conclusions. Purposive sampling was used to efficiently target potential participants from the specified group of interest. Our use of purposive sampling resulted in a sample of 18 participants from a relatively small number of organizations. One question to ask therefore is whether our approach has biased us towards identifying similar respondents, which is a possibility when using this method [16]. A more diverse sample might have been desirable; however, consistency in data contributed from participants suggests that additional data might not have changed the results and conclusions.

It might be expected that purposive sampling used in this manner (i.e., using personal direct contacts and networks) would result in respondents with similar views to the investigators. This was not observed, however. The generally low awareness of the capabilities of ML, and moreover the aims of integration of ML into EBM, indicates that participants most likely did not hold similar opinions to the researchers.

One clear skew in the resulting sample was the inclusion of only 28% male participants. Despite this, the views of participants did not appear to vary according to gender. It is possible that this balance is representative of the current balance in the field of guideline development; for example, according to data available from NICE, they employ 68.63% women and 31.37% men [17].

An additional result of the sampling technique was the potential over-representation of Australian professionals. Five participants, however, had direct experience in low-resource settings and/or originated from countries other than their current base, broadening the potential perspectives for this analysis. Nevertheless, any use of these results should be tempered by awareness of the strong Australian, UK, and US representation in the data collected.

### Current state on the adoption curve

**Fig. 2:**
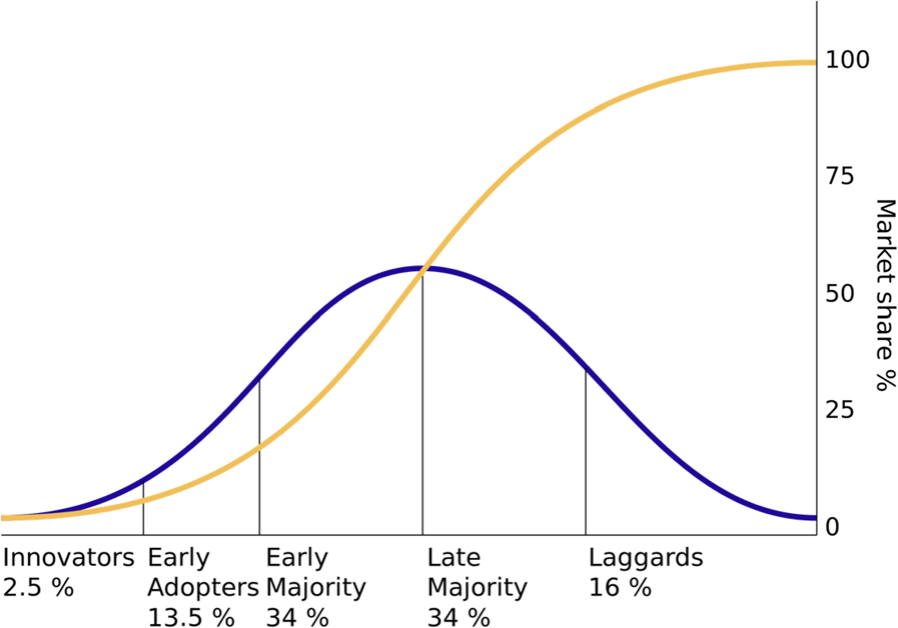
Diffusion of Innovations adoption curve and market share

Finally, as previously outlined in the “Background” section, *Diffusion of Innovations* describes the typical categories of innovators (personas) and provides an approximation of the expected proportions of each category. These are shown in Fig. 2. As time progresses, successive groups will adopt a given innovation, until a critical mass of the market share is reached. In addition to the five categories shown, non-adopters are sometimes added as a sixth category.

The finding that many of the participants perceived themselves to be inexperienced with automation in the context of evidence synthesis raises the question of where the field currently resides within this adoption curve. The evidence of this study suggests that the field is in very early stages of broader adoption (i.e., innovators) with only a small minority taking on the use of this new technology.

Once a later stage has been reached, subsequent studies may well return different results. For example, the case from participant 11 in *Observability: need for evidence*, while certainly an outlier in the context of this study, may fall under an innovator or early adopter persona while other participants may fall under late majority or laggards. Additional analysis and/or data collection using persona categories as the deductive framework could build upon the results of this study. However, until a later stage of diffusion is reached, it may be difficult to find sufficient contributors within each category.

### Suggestions for future research

While guideline developers are a crucial group within the field of evidence-based medicine, they are far from the only one. As mentioned in the “Background” section, patients, caregivers, consumers, healthcare professionals, and researchers are also key stakeholders in evidence synthesis. Therefore, it would be logical to repeat this study with different population groups. Systematic reviewers could be considered a high priority for consultation, as these individuals will be using automation software directly, as opposed to guideline developers who act as gatekeepers of the output (i.e., health evidence) of such software.

Patient stakeholders are also an important group to consult. Patients are often involved in guideline panels, and there have been recent pushes to include more consumers and patients in the development of health guidance [18]. Health guidelines should ultimately aim to benefit patients and the community, and organizational mission statements often (and rightly) include statements about patient transparency and empowerment. Finally, policymakers should also be examined, as they were identified by some of the participants in this study as fellow stakeholders in the process of creating guidelines and whose values influence the practices of evidence synthesis.

Future research should be prioritized and proceed in parallel to the forms of validation highlighted by participants as crucial to their decision making. Select examples of automation have long been available for evidence synthesis, and several prominent organizations are encouraging automation uptake. Despite this reality, they are not being integrated into workflows at a large or even a medium scale. The data from this study show hesitation from a key stakeholder group, and additional data relating to other user stakeholders will be helpful in identifying barriers and facilitators for these groups.

## Conclusions

Analyzed via the lens of the Diffusion of Innovations framework, the results of this study strongly demonstrate that *Compatibility* with professional cultural values is the most significant consideration for guideline developers in the potential adoption of automation. Participating guideline developers identified increased availability of person-time as a primary *Relative Advantage*, and desired rigorous validation (*Observability*) to occur both prior to adoption and on an ongoing basis. A lack of knowledge around ML among participants is a contributing contextual factor to the slow uptake of automation, along with a generalized anxiety towards relinquishing human control to a computer. This contextual factor means that future studies may return different results if and when the evidence synthesis field reaches a later stage in the adoption curve. The data demonstrated an inaccurate perception that nuanced human judgment is likely to be removed from evidence synthesis by automation.

The creation and dissemination of empirical evidence that systematically demonstrates automation’s alignment with the values and standards of guideline development and EBM should therefore be prioritized. In addition, disseminated evidence and communications around automation tools may benefit from focusing on the combination of human and ML effort, rather than the replacement of human insight. Finally, proponents should prioritize communication of transparency in automation methods and on strengthening automation competency and familiarity among EBM professionals.

## Supplementary Information

**Additional file 1.** COREQ (COnsolidated criteria for REporting Qualitative research) Checklist.

**Additional file 2.** Interview Instrument.

## Data Availability

The COREQ checklist and the interview instrument are available as supplementary material. Anonymized data will be securely stored according to the UCL Institute of Education guidance and may be made available upon reasonable request.

## Abbreviations

EBM: Evidence-based medicine
ICASR: International Collaboration for the Automation of Systematic Reviews
ML: Machine learning
NHMRC: National Health and Medical Research Council (Australia)
NICE: National Institute for Health Care and Excellence (UK)
WHO: World Health Organization
