# Supplementary material for: The views of health guideline developers on the use of automation in health evidence synthesis
Source: Syst Rev. 2021 Jan 8;10:16. doi: 10.1186/s13643-020-01569-2 (PMC7796617; doi:10.1186/s13643-020-01569-2)
Supplement: Supplementary file 2 — Additional file 2. Interview Instrument. [file 13643_2020_1569_MOESM2_ESM.pdf]

Screening: Can you describe your experience and role in guideline development?

- Must be involved in the decision-making process of whether a piece of evidence is accepted into the guideline or not. If participant is determined early in the interview to not fit this criterion, interview will be wrapped up.

1. What do you view as the goal of guideline development?
2. What do you view as the end goal of evidence synthesis (including systematic reviews)?
3. What method do you or does your organisation use to collect evidence?
4. Are there any limitations on the type of evidence you will accept?
5. What do you think are the best methods for production of evidence?
6. What is your opinion of machine-learning in relation to evidence synthesis?
7. What is your opinion of crowd-sourcing in relation to evidence synthesis?
8. What do you feel the opinion of the guideline developer community as a whole is, in relation to machine learning and crowd?
9. Would you be more likely or less likely to accept the conclusions of a systematic review which had used automation in its protocol?
10. What factors might influence this decision?
11. In your ideal world, what would the future look like in terms of use of automation for evidence synthesis? What sorts of capabilities would these technologies have, and what would they not have?
